# Rare Case of Germline *GATA2* ‐Deficiency With Merkel Cell Carcinoma and Acute Myeloid Leukemia

**DOI:** 10.1002/cnr2.70068

**Published:** 2024-11-29

**Authors:** SooHo Yu, Min‐Seung Park, Gyu Yeong Kim, Junhun Cho, Chul Won Jung, Hee‐Jin Kim, Hyun‐Young Kim

**Affiliations:** ^1^ Department of Laboratory Medicine and Genetics, Samsung Medical Center Sungkyunkwan University School of Medicine Seoul Korea; ^2^ Department of Laboratory Medicine, Kangbuk Samsung Hospital Sungkyunkwan University School of Medicine Seoul Korea; ^3^ Department of Pathology and Translational Genomics, Samsung Medical Center Sungkyunkwan University School of Medicine Seoul Korea; ^4^ Division of Hematology‐Oncology, Department of Medicine, Samsung Medical Center Sungkyunkwan University School of Medicine Seoul Korea

**Keywords:** acute myeloid leukemia, *GATA2*‐defieciency, Merkel cell carcinoma

## Abstract

**Background:**

Germline *GATA2*‐deficiency usually manifests as immunodeficiencies and myeloid neoplasms and sometimes with dermatological diseases, including warts, panniculitis, and skin cancers.

**Case:**

We report a 36‐year‐old woman with germline *GATA2*‐deficiency who developed Merkel cell carcinoma followed by acute myeloid leukemia. Molecular analysis revealed a germline *GATA2* S447R variant, not reported from the previous reported case, suggesting a potential association with Merkel cell carcinoma.

**Conclusion:**

This case broadens the spectrum of solid cancers linked to *GATA2*‐deficiency, emphasizing the need for considering primary immunodeficiency in young patients with myeloid neoplasms or rare skin cancers, facilitating early detection and treatments.

## 1 Introduction

*GATA2* is a zinc‐finger transcription factor that is critical for hematopoiesis. Patients with germline *GATA2*–deficiency may present immunodeficiencies and myeloid neoplasms, including DCML deficiency (combined deficit of dendritic cell, monocyte, B and NK lymphoid cells), MonoMAC (monocytopenia and mycobacterial infection) with associated recurrent infections, myelodysplastic neoplasm (MDS), acute myeloid leukemia (AML), and chronic myelomonocytic leukemia [1, 2]. Dermatological diseases including warts and panniculitis, also frequently occur and the age of the first onset ranges from 6 to 40 years old. There are even several reports of skin cancers developed in *GATA2*–deficiency patients; basal cell carcinoma, squamous cell carcinoma, and malignant melanoma [1].

Merkel cell carcinoma (MCC) is a rare and aggressive form of skin cancer, typically presenting as a painless nodule with varying color in the head and neck region [3, 4]. Herein, we report the case of a patient with germline *GATA2*–deficiency who first developed MCC followed by AML.

## 2 Case

**FIGURE 1:**
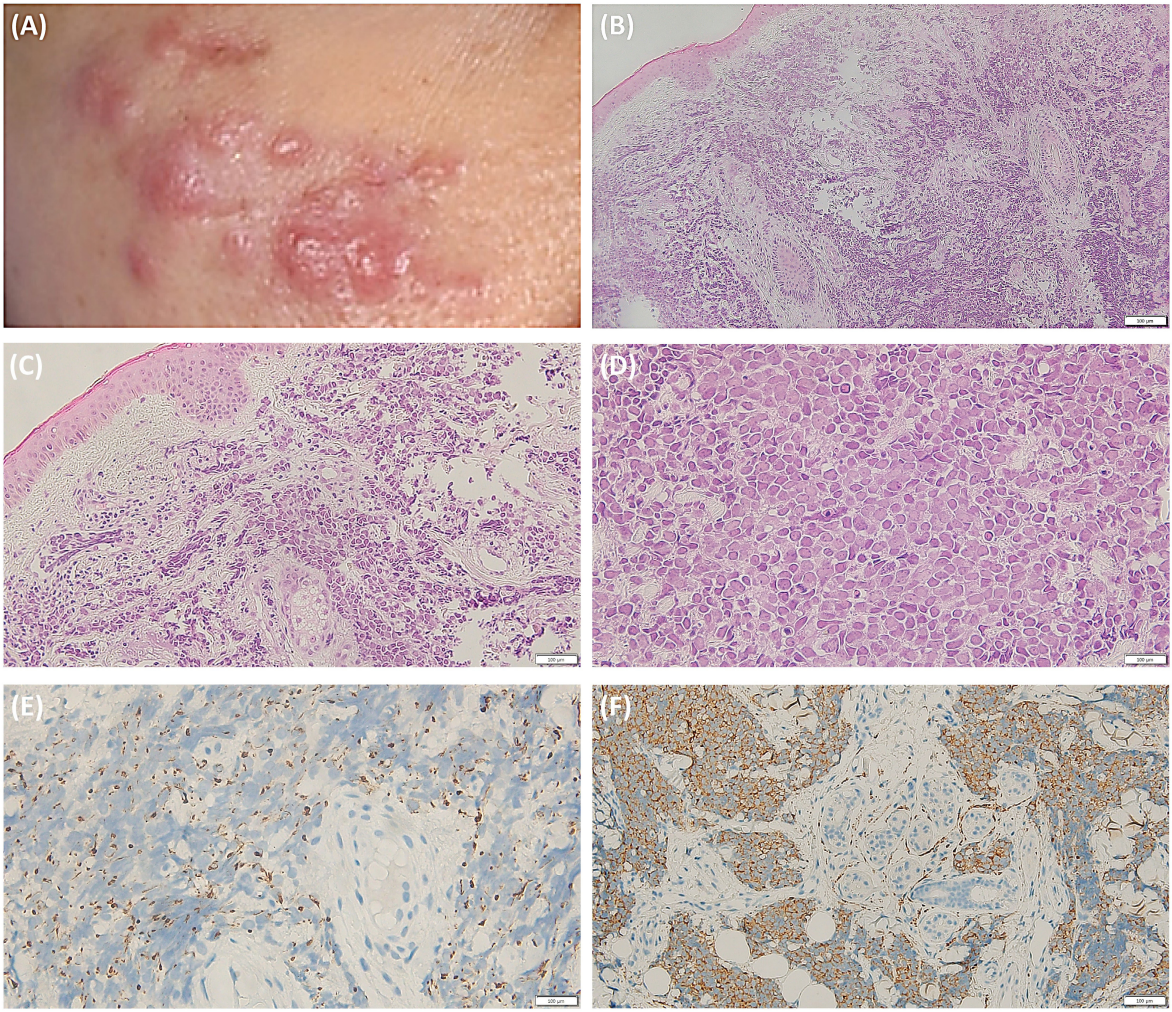
(A) Skin lesion of Merkel cell carcinoma (MCC). (B) Skin biopsy (hematoxylin and eosin staining, ×100) with diffusely infiltrative MCC, (C) with pure neuroendocrine morphology (×200), and (D) mature neuroendocrine carcinoma with salt and pepper nuclear chromatin pattern and indistinct nucleoli (×400). The neoplastic cells were (E) CK20‐positive with a dot‐like paranuclear pattern (×400) and (F) synaptophysin–positive with a diffuse cytoplasmic pattern (×200). Scale bars are 100 μm.

A 36‐year‐old woman visited Samsung Medical Center with a 4–month history of a growing right–sided cheek skin lesion on October 2021 (Figure 1A). The skin biopsy revealed a diffusely infiltrative neoplasm with pure neuroendocrine morphology. The neoplastic cells were CK20‐positive and synaptophysin‐positive (Figure 1B–F). The patient was diagnosed with MCC and underwent wide excision and defect coverage. No evidence of metastatic lesions was observed, and the patient did not receive cytotoxic chemotherapy after skin excision. A complete blood count (CBC) at the time of admission revealed pancytopenia: hemoglobin 10.9 g/dL; white blood cells 1.68 × 10^9^/L and absolute neutrophil count (ANC) 0.84 × 10^9^/L; and platelets 125 × 10^9^/L.

**FIGURE 2:**
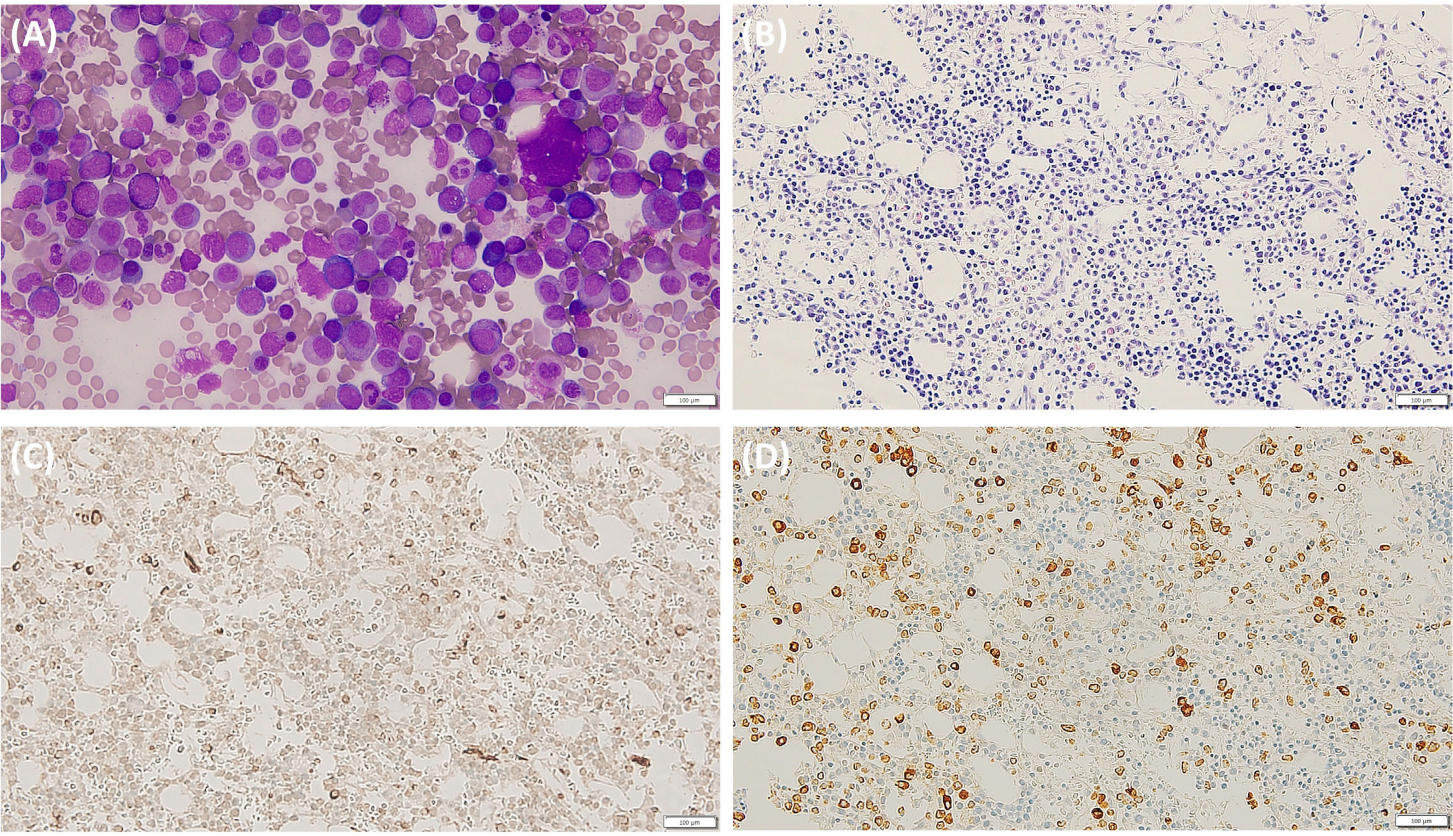
Bone marrow (BM) aspiration and biopsy. Increased leukemic blasts were observed in (A) BM aspiration (Wright–Giemsa stain, ×400) and (B) biopsy (hematoxylin and eosin staining, ×100). Leukemic blasts were (C) CD34–positive and (D) MPO–positive (×200). Scale bars are 100 μm.

In September 2022, the patient visited the hospital with menorrhagia and easy bruising. A CBC revealed aggravated pancytopenia: hemoglobin 7.0 g/dL; white blood cells 1.2 × 10^9^/L (ANC 0.7 × 10^9^/L); and platelets 83 × 10^9^/L. A bone marrow (BM) study revealed 20.8% blasts with decreased and dysplastic trilineage hematopoietic cells (Figure 2A–D). Flow cytometry revealed blasts expressing CD34 and positive for CD13, CD33, and CD117; weakly positive for MPO; and negative for other lineage–specific markers, including surface and cytoplasmic CD3, CD19, CD10, and cytoplasmic CD79a, a finding consistent with AML. Cytogenetic analysis revealed a complex karyotype: 42,XX,del(3)(q12),‐5,add(7)(q32),‐11,‐13,‐16,‐17,del(20)(q11.2),+mar,inc [3]/46,XX[17], and AML with myelodysplasia‐related changes was diagnosed. Moreover, targeted next‐generation sequencing (NGS) for 46 AML‐related genes identified three variants: *GATA2* S447R (NM_0032638.4:c.1341C > A; variant allele frequency (VAF), 49%), *TP53* R248Q (NM_000546.5:c.743G > A; 12%), and *STAG2* R451fs*12 (NM_001042749.2:c.1351dup; 30%). *GATA2* S447R was confirmed as germline likely pathogenic variant in targeted Sanger sequencing using the patient's cultured skin fibroblast. The patient received induction chemotherapy with a standard dose of cytarabine and idarubicin, achieved complete remission, and was scheduled to undergo allogeneic hematopoietic stem cell transplantation. However, after consolidation chemotherapy, the patient failed to recover from neutropenia and developed an anorectal abscess and pulmonary mucormycosis. The patient's condition deteriorated and she expired 5 months after the diagnosis of AML.

## 3 Discussion

The natural course of *GATA2*‐deficiency is highly variable with wide range of disease onset and clinical presentations. The majority of *GATA2*‐deficiency patients demonstrate major infections, MDS, or AML, and when dermatologic diseases are present, they often serve as an early indicator [1]. To our knowledge, this is the second report of MCC with germline *GATA2*–deficiency, following Crall et al. [5], who reported MCC in a 55–year–old woman with *GATA2*–deficiency and neurofibromatosis 1, but did not specify the *GATA2* variant information. Our patient was diagnosed with MCC at a relatively early age, without underlying diseases and comorbidities related to *GATA2*–deficiency. MCC is a rare but highly aggressive carcinoma in the dermis and/or subcutis with neuroendocrine features and is positive for chromogranin–A, synaptophysin, and cytokeratin 20 [6, 7]. MCC is prevalent in immunocompromised individuals, older adults, those with autoimmune diseases under immunosuppressive treatment, or Merkel cell polyomavirus infection, and those exposed to chronic ultraviolet light [6, 8, 9, 10]. Germline *GATA2* variants induce primary immunodeficiency and increase susceptibility to infections due to immune dysfunction, which is probably responsible for the high incidence of solid cancers [11]. Primary immunodeficiency induced by *GATA2*‐deficiency might have played a major role in the development of MCC, supporting a possible association between *GATA2*–deficiency and MCC.

The *GATA2* S447R is far away from C‐finger domain of *GATA2*, but is expected to alter GATA2 levels through augmentation of the C‐terminal transactivation domain or disruption of the critical ubiquitination region [12]. The previously reported symptomatic patients with the *GATA2* S447R were diagnosed with myeloid neoplasms (MDS, AML) and aplastic anemia, but none presented solid cancers [12, 13]. The previous 9 patients shared recurrent somatic mutations (*ASLX1*, *DNMT3A*) and karyotype abnormality (monosomy 7), and all survived after receiving hematopoietic stem cell transplantation, except one case who died of infection after transplantation. The discrepant prognosis between the previous report and our patient would have been due to the difference of accompanying somatic mutations and karyotype abnormalities (*ASLX1*, *DNMT3A*, and monosomy 7 vs. *TP53*, *STAG2*, and complex karyotype).

In conclusion, we provide evidence supporting an association between *GATA2*–deficiency and MCC. The identification of the germline *GATA2* S447R variant, previously unreported in association with MCC, suggests a potential link and further investigation. In young MCC patients or myeloid neoplasm patients with a history of rare skin cancer, the possibility of primary immunodeficiency must be considered, such as *GATA2*–deficiency, to allow early detection and the choice of treatment strategies, potentially improving patient outcomes. This case broadens the spectrum of solid cancers linked to *GATA2*‐deficiency and emphasizes the importance of considering underlying genetic causes in atypical presentations of cancer.

## Author Contributions

**SooHo Yu:** writing – original draft; writing – review and editing; investigation; visualization. **Min‐Seung Park:** writing – original draft; investigation; conceptualization. **Gyu Yeong Kim:** resources; visualization. **Junhun Cho:** resources. **Chul Won Jung:** resources. **Hee‐Jin Kim:** supervision; resources. **Hyun‐Young Kim:** writing – review and editing; conceptualization; supervision.

## Consent

This study was approved by the Institutional Review Board (IRB) of Samsung Medical Center, Seoul, Korea (2023‐02‐079), and the requirement for informed consent was waived in accordance with Article 16, Paragraph 3 of the Bioethics and Safety Act of the Republic of Korea. The IRB granted a wavier for obtaining consent from the patient's next of kin, due to the time elapsed since the patient's death, with minimal risk to the study's ethical standards or integrity.

## Conflicts of Interest

The authors declare no conflicts of interest.

## Data Availability

The data that support the findings of this study are available from the corresponding author upon reasonable request.
